# A novel flow cytometry-based assay for the quantification of antibody-dependent pneumococcal agglutination

**DOI:** 10.1371/journal.pone.0170884

**Published:** 2017-03-13

**Authors:** Marrit N. Habets, Saskia van Selm, Christa E. van der Gaast—de Jongh, Dimitri A. Diavatopoulos, Marien I. de Jonge

**Affiliations:** Laboratory of Pediatric Infectious Diseases, Department of Pediatrics, Radboud Institute for Molecular Life Sciences, Radboud university medical center, Nijmegen, The Netherlands; Instituto Butantan, BRAZIL

## Abstract

The respiratory pathogen *Streptococcus pneumoniae* is a major cause of diseases such as otitis media, pneumonia, sepsis and meningitis. The first step towards infection is colonization of the nasopharynx. Recently, it was shown that agglutinating antibodies play an important role in the prevention of mucosal colonization with *S*. *pneumoniae*. Here, we present a novel method to quantify antibody-dependent pneumococcal agglutination in a high-throughput manner using flow cytometry. We found that the concentration of agglutinating antibodies against pneumococcal capsule are directly correlated with changes in the size and complexity of bacterial aggregates, as measured by flow cytometry and confirmed by light microscopy. Using the increase in size, we determined the agglutination index. The cutoff value was set by measuring a series of non-agglutinating antibodies. With this method, we show that not only anti-polysaccharide capsule antibodies are able to induce agglutination but that also anti-PspA protein antibodies have agglutinating capabilities. In conclusion, we have described and validated a novel method to quantify pneumococcal agglutination, which can be used to screen sera from murine or human vaccination studies, in a high-throughput manner.

## Introduction

Nasopharyngeal colonization with the respiratory pathogen *Streptococcus pneumoniae* is a prerequisite for the development of pneumococcal disease. Following dissemination of bacteria to the ear, lung, bloodstream or brain, otitis media, pneumonia, sepsis or meningitis may develop, respectively. Several mucosal defense mechanisms, such as antibody-mediated opsonization and opsonophagocytosis by phagocytes, have been suggested to be important in the reduction or complete prevention of colonization [1,2]. Recently, Roche *et al*. (2015) showed that the presence of agglutinating antibodies on the mucosal surface plays an important role in the prevention of pneumococcal colonization in a mouse model of colonization and transmission [3]. The agglutinating properties of antibodies raised against novel vaccine candidates might therefore be predictive for efficacy, and would be an important parameter to include in clinical trials.

The agglutinating properties of antibodies against *S*. *pneumoniae* have long been known. In 1902 Neufeld described agglutination of pneumococci with specific antisera, visible as capsular swelling and clumping of bacteria, which became known as the quellung reaction [4]. Classification of pneumococci by specific serological reactions was described in 1913 [5,6], and the identification of new serotypes followed over the years, leading to the description of 80 distinct serotypes in 1960 [7]. Since then, the quellung reaction using polyclonal rabbit antisera has been the ‘gold standard’ for serotyping. Capsular swelling, which is usually accompanied by agglutination, is typically visualized microscopically. Latex agglutination or slide agglutination is viewed macroscopically. [8,9]. While the determination of an agglutination titer for serum against several bacteria has been described using a tube or well agglutination test, where a titer is determined using serially diluted serum that is mixed with a constant quantity of bacteria [10], variability in individual interpretation of results makes it difficult to standardize this method. In addition, this method is relatively time consuming and therefore not very suitable for high-throughput use.

Mucosal protection against colonization by agglutinating capsule-specific antibodies is thought to be mediated predominately by immunoglobulin G. Although IgA1 antibodies, which represent the most abundant immunoglobulin subclass present on the airway mucosa, can also induce bacterial aggregates, the expression of IgA1 protease by *S*. *pneumoniae* has been shown to negate this effect [3]. The pneumococcal conjugate vaccines (PCVs) induce high amounts of systemic IgG against several different types of capsular polysaccharides. Due to active transport to the mucosal surface via the neonatal Fc receptor, these antibodies also provide protection against pneumococcal colonization [11,12]. Recent studies have shown that agglutination by anti-pneumococcal IgG antibodies contributes to protection against pneumococcal colonization [3,13].

Since the agglutinating effect of antibodies has shown to be an important factor in the protection against pneumococcal colonization, there is a clear need for adequate methods to assess and quantify this antibody functionality. However, to date, there is no standardized method to measure pneumococcal agglutination. Here, we developed a high-throughput method to screen serum samples for their agglutinating potential of various pneumococcal strains, using flow cytometry. Using this novel method, we assessed the agglutinating potential of both capsule-specific antibodies and antibodies generated against the pneumococcal surface protein A (PspA).

## Materials and methods

### Pneumococcal strains

The *Streptococcus pneumoniae* serotype 4 strain TIGR4 [14] and the serotype 19F strain EF3030 [15] were used in agglutination experiments with anti-capsular antibodies. Non-encapsulated strains were used in experiments with anti-PspA antisera. The non-encapsulated derivative of TIGR4 (PspA clade 3) and G54 (PspA clade 4) and construction of these mutants were described before [16]. Other non-encapsulated strains differing in PspA clades [17] and used in agglutination with anti-PspA sera were constructed with primer pair FI4 and PE21 [18] as described before [16]. These capsule locus deletion mutants were constructed of strains EF3030 (clade 1; [15]), PBCN0226 (clade 2; [19]), BHN100 (clade 3; [20]), and PBCN0117 (clade 5; [19]). A PspA deletion mutant of the non-encapsulated TIGR4 strain was constructed by allelic exchange using a spectinomycin resistance cassette. Overlap extension PCR was applied to insert the spectinomycin resistance cassette between the flanking regions of the *pspA* gene. The two flanking regions and the spectinomycin resistance cassette of plasmid pR412 [21] were PCR-amplified with the CvdG_SP_0117-pspA_L1/L2 (GCAAGTTGTTGCATCGTAGC/ CCACTAGTTCTAGAGCGGCTGAGACGTAACAAAACC), CvdG_SP_0117-PspA R1/R2 (CTCATGGTAAGTCTCCCATC/ GCGTCAATTCGAGGGGTATCAATGCCAATGGTGAATGGG) and (GCCGCTCTAGAACTAGTGG/ GATACCCCTCGAATTGACGC) primer pairs. The overlap extension PCR product was transformed in the unencapsulated TIGR4 strain, directed mutants were selected by selective plating with 150 μg/ml spectinomycin and correct integration was confirmed by PCR with primer CvdG_SP_0117-pspA_C (CAACAAGCGCCACATCATTC). The bacteria were grown in Todd Hewith Broth (Becton Dickinson), supplemented with 5% Yeast extract (Becton Dickinson) (THY medium) to mid-log phase and stored at -80°C in THY medium supplemented with 15% glycerol. The number of viable bacteria in frozen stocks was enumerated by three times independent plating on blood agar plates (Becton Dickinson).

### Sera

Polyclonal rabbit serum against serotype 4, serotype 14 and serotype 19F (factor serum 19**b**) were used (Statens Serum Institute, Denmark). Polyclonal sera against PspA were used from an animal study in which 8 week old BALB/C mice (Charles River Laboratories, Germany) were immunized intranasally with PspA adjuvanted with Cholera Toxin subunit B (CTB). Mice were immunized intranasally under inhalation anesthesia (isoflurane) with 10 μg His-tagged PspA from TIGR4 (kindly provided by Mucosis B.V., Groningen, The Netherlands), 4 μg CTB (Sigma) and PBS in a 10 μl volume three times, with 2 week intervals. The mice in this vaccination study were challenged with 10^6^ CFU TIGR4 in PBS in a 10 μl volume divided over both nares of anesthetized mice. Four days post inoculation euthanasia was performed by terminal bleeding under inhalation anesthesia (isoflurane), followed by cervical dislocation. Blood was centrifuged at 7000 x g and serum was stored at -20°C. Serum from mice immunized with CTB alone in PBS was used as negative control (mock). The animal experiment was approved by the Radboud university medical center Committee for Animal Ethics (approval number RU-DEC 2012–175) and conducted in accordance with the relevant Dutch legislation.

### Agglutination assay

Pneumococci were diluted in THY from -80°C stocks. Experiments were performed using non-coated V-bottom 96-well plates (Falcon). For each reaction, 10^5^ colony forming units (CFU) were incubated in duplicate with various serum dilutions for 1 hour in a total volume of 50 μl for anti-polysaccharide agglutination and 10 μl for anti-PspA agglutination, at 37°C and 5% CO_2_. Bacteria were then gently fixed by adding paraformaldehyde to a final concentration of 1%. After fixation the samples were either analysed by phase contrast microscopy or diluted with PBS (Lonza) for direct analysis by flow cytometry. All experiments were performed in at least 2 but mostly 3 independent experiments.

### Analysis of agglutination by flow cytometry

Bacteria were measured on a BD LSRII Flow Cytometer (BD Biosciences) and BD FacsDiva Software 6.1 (BD Biosciences, San Jose, CA, USA). Pneumococci were gated and a dot-plot was generated by forward scattering (FSC-A) on the x-axis and side scattering (SSC-A) on the y-axis. To enable reproducible measurements, PMT voltages and threshold were adjusted gated on negative control bacteria, and the speed of the flow was set at 0.5 μl/ sec for all measurements. The cutoff value for FSC positive agglutination was set using the negative control without serum. All samples were analyzed in duplicate and between 5000–100000 events were acquired for each measurement. Results were analysed by FlowJo version vX.07 (FlowJo, TreeStar, Inc, Ashland, U.S.A.).

### Phase contrast microscopy

Bacterial aggregates were visualized by phase contrast microscopy. Bacterial samples were transferred to glass slides, sealed with nailpolish and incubated overnight at room temperature before microscopic imaging. Images were acquired at a magnification of 6300x using a Leica DM600 B microscope.

## Results

### Quantification of capsular antibody-dependent pneumococcal agglutination by flow cytometry

**Fig 1:**
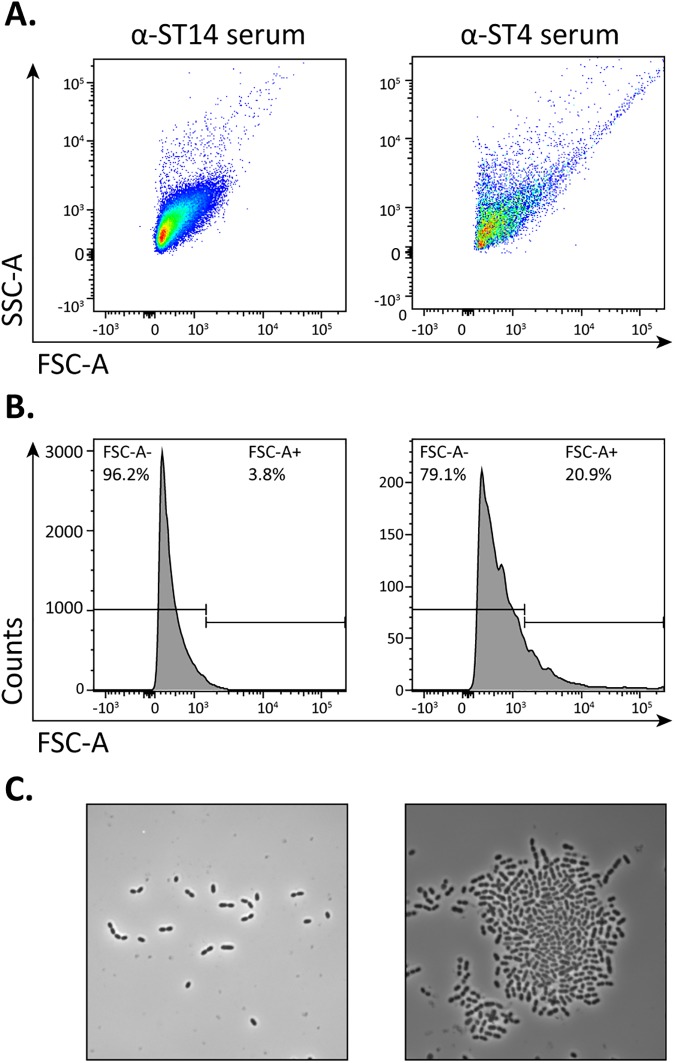
Agglutination of *S*. *pneumoniae* by anti-capsule antibodies can be detected by flow cytometry. *S*. *pneumoniae* TIGR4 were incubated with a serotype specific polyclonal rabbit antiserum (serotype 4; α-ST4; right panel) and with rabbit antiserum against a heterologous serotype (serotype 14; α-ST14; left panel). Agglutination was assessed by flow cytometry and is represented as dot plots of the FSC-A vs the SSC-A **(A)** and histograms of the FSC-A signal **(B)** and by phase contrast microscopy **(C)**.

To determine if agglutination can be measured by flow cytometry, we compared agglutinated with non-agglutinated pneumococci. *S*. *pneumoniae* TIGR4 was incubated with agglutinating serotype 4-specific (ST4) polyclonal rabbit antiserum as well as with a non-agglutinating heterologous control antiserum against serotype 14 (ST14). Following flow cytometry analysis, we found that a large proportion of the bacteria incubated with ST4-specific serum had increased on both the FSC and SSC axis, as compared to the bacteria incubated with ST14-specific serum (Fig 1A & 1B). In addition, the number of events measured in the FSC channel, which is representative of the number of particles detected by the flow cytometer, was reduced in the presence of agglutinating antibodies. This suggests the formation of bacterial aggregates, which was confirmed by phase contrast microscopy analysis (Fig 1C).

**Fig 2:**
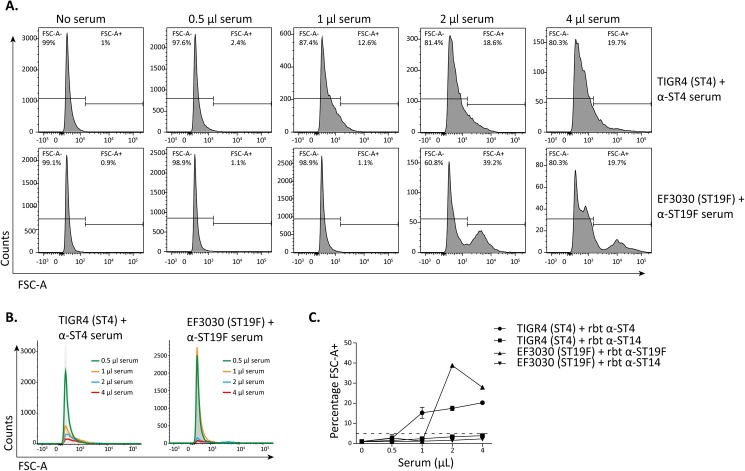
Pneumococcal agglutination by anti-capsule antibodies can be quantified using flow cytometry. Agglutination with two pneumococcal strains (TIGR4 and EF3030) incubated with different concentrations of serotype-specific rabbit antiserum (serotype 4 or serotype 19F) and rabbit antiserum against a heterologous serotype (serotype 14) (performed in duplicate). Individual histograms of a representative measurement are shown for the forward scatter of TIGR4 and for EF3030 following incubation with type-specific antiserum **(A),** overlays of the different concentrations of type-specific antiserum are shown **(B)** and all data (type-specific and heterologous antiserum) is summarized in a single graph **(C)**. The dashed line represents the agglutination cutoff value.

In order to quantify pneumococcal agglutination, we incubated *S*. *pneumoniae* TIGR4 (serotype 4) or *S*. *pneumoniae* EF3030 (serotype 19F) with serial dilutions of serotype-specific anti-capsular antibodies, i.e. anti-ST4 and anti-ST19F, or with heterologous anti-serotype 14 serum. Flow cytometry analysis showed a clear increase for both TIGR4 and EF3030 in the percentage of FSC-positive cells with increasing concentrations of serotype-specific antibodies (Fig 2A). This was accompanied by a decrease in the number of events in the FSC channel (Fig 2B). Neither TIGR4 nor EF3030 showed an increase in FSC following incubation with anti-serotype 14 serum (Fig 2C). The FSC values of these control samples always remained below 5%, which was therefore used as the cutoff for positivity. We termed the percentage of FSC-positive bacteria the agglutination index. For both TIGR4 and EF3030, a clear increase in the agglutination index could be observed in the presence of increasing concentrations of type-specific serum (Fig 2C). Samples were treated with paraformaldehyde before measurement on the flow cytometer, which did not affect the agglutination index.

### Antibodies against the pneumococcal protein PspA are capable of inducing agglutination

**Fig 3:**
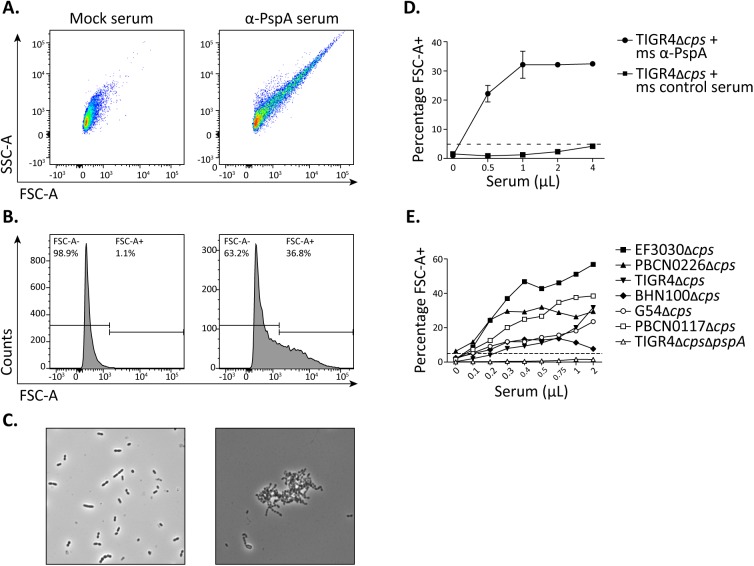
Antibodies against the pneumococcal protein PspA are capable of inducing agglutination. An *S*. *pneumoniae* TIGR4 capsule mutant strain was incubated with increasing amounts of serum from mice that had been mock-vaccinated (control serum) or that had been vaccinated with PspA (α-PspA serum) (performed in duplicate). Agglutination was visualized by flow cytometry as dot plots of the FSC-A and SSC-A signal **(A)**, as histogram of the FSC-A signal **(B)** or with phase contrast microscopy **(C).** Flow cytometry data is summarized in a single graph for both the anti-PspA serum and the control serum **(D)**. Nonencapsulated pneumococcal strains with PspA sequences from clades 1–5 (EF3030: clade 1, PBCN0226: clade 2, TIGR4: clade 3, BHN100: clade 3, G54: clade 4, PBCN0117: clade 5), as well as a PspA mutant strain (TIGR4Δ*cps*Δ*pspA*) were incubated with increasing amounts of mouse anti-PspA serum **(E)**. The dashed line represents the agglutination cutoff value.

Next, we sought to investigate if antibodies generated against a pneumococcal protein antigen known to confer mucosal protection, would also be capable of inducing agglutination. To do so, we made use of antiserum against pneumococcal surface protein A (PspA) from a mouse vaccination study. An *S*. *pneumoniae* TIGR4 capsule mutant strain (TIGR4Δ*cps*) was incubated with serial dilutions of anti-PspA or control mouse serum. Samples were then analysed by flow cytometry. Similar to the anti-capsular serum used before, anti-PspA antibodies also induced a shift in the FSC and SSC of the bacterial population (Fig 3A and 3B). Analysis of the samples by phase contrast microscopy confirmed that incubation of pneumococci with PspA-specific serum induced the formation of aggregates, which did not occur in the presence of control serum (Fig 3C). Incubation with different concentrations of anti-PspA serum showed that the agglutination index reached a plateau when more than 1 μl of serum was added per 10^5^ bacteria (Fig 3D). The anti-PspA serum was obtained from mice vaccinated with recombinant PspA representative of PspA clade 3 [14]. To determine if serum against this particular PspA type was also able to induce agglutination of pneumococcal strains with other PspA alleles, non-encapsulated pneumococcal strains with PspA sequences from clade 1, 2, 3, 4 and 5 were incubated with increasing amounts of anti-PspA serum. As a negative control, a PspA mutant strain (TIGR4Δ*cps*Δ*pspA*) was used. Fig 3E shows that the anti-PspA serum induces agglutination for pneumococcal strains with PspA sequences from all the different clades. Nevertheless, major differences were observed between the agglutination index of pneumococcal strains belonging to different PspA clades, with clade 1 showing the highest agglutination index, and the BHN100 strains with a PspA from clade 3 having the lowest agglutination index. As expected, the control PspA mutant strain did not agglutinate.

## Discussion

Antibody-mediated agglutination of pneumococci has recently been shown to be an important factor in the prevention of pneumococcal colonization [3]. While bacterial agglutination may be analyzed using methods such as light microscopy, they are generally not very suitable for quantification and/or high-throughput analysis. Thus, we present a novel flow cytometry-based assay to quantify the pneumococcal agglutinating capacity of serum. This assay can be easily adapted to incorporate other strains and/or other bacterial species and allows screening of serum samples in a high-throughput manner. Finally, we show that both anti-capsule and anti-protein antibodies are able to induce agglutination.

In this manuscript, we show that antibody-induced agglutination correlated with a shift in the FSC and SSC properties of the bacterial particles, as measured by flow cytometry. These findings were validated by light microscopy. The shift in FSC represented the agglutination index, i.e. the percentage of bacteria with a positive FSC signal. The actual percentage of agglutinating bacteria is probably higher, since agglutinates might have fallen apart due to the shearing force that is exerted in the FACS capillaries. In addition, bacterial aggregates are detected as single particles by the flow cytometer, whilst in reality they consist of multiple bacteria clumped together. The latter is also reflected in the number of events that were measured in the FSC channel, the total of which was much lower for the agglutinated samples as compared to the non-agglutinated samples. The agglutination index is therefore influenced by several factors; the actual number of agglutinating bacteria, the affinity of the antibodies, and the concentration of antibodies relative to the bacteria. Measurement of the absolute number of bacteria with for example a DNA stain, could provide more insight in the actual percentage of agglutinating pneumococci.

When comparing the agglutination index of different samples, we suggest to use a constant amount of serum. However, it is also possible to use other methods such as the determination of an endpoint titer. The cutoff value for positive agglutination was set at 5%, which is based on data obtained with non-heterologous serotype specific serum. The cutoff value at which agglutination leads to protection against colonization remains to be studied. The current method has been further validated on human samples in a larger study, where post-vaccination nasal washes from a human pneumococcal challenge model mediated increased pneumococcal agglutination as compared to nasal washes collected before vaccination [13].

All sera that were used in this study are likely to contain not only IgG, but also IgA (primarily IgA1). However, it has been shown that IgA1 has no agglutinating effects in IgA1 protease-producing pneumococcal strains [3]. In addition, since all sera used in this study were raised by multiple booster vaccinations during a period of at least 9 weeks, class switching will have taken place, which means that levels of IgM are negligibly low.

Before the introduction of PCVs, pneumococcal carriage rates typically declined after the age of 3 [2], which is thought to be caused by the establishment of broad natural immunity to conserved pneumococcal proteins and capsular antigens [22–25]. The high amounts of systemic IgG that are induced by PCVs are indeed correlated to protection against colonization [11,12] and agglutination has recently been suggested to be the main mechanism for this protection. However, the role of antibodies targeting pneumococcal proteins is not yet clear [3,13]. We and others have shown that intranasal vaccination with the pneumococcal surface protein A can protect mice, not only against systemic infection and pneumonia, but also against nasopharyngeal colonization [26–28]. This vaccine-induced protection has frequently been attributed to PspA-specific cellular immunity. Conversely, the contribution of antibody-dependent agglutination has, to the best of our knowledge, not been investigated before. Our findings show that murine antibodies against PspA have the ability to agglutinate a range of pneumococcal strains belonging to different PspA clades. Our rationale for using non-encapsulated strains was because pneumococci are known to downregulate their capsule [29,30]. However, capsule expression is difficult to control. When carriage isolates are cultivated in the laboratory they likely obtain a thick capsule, similar to what is observed when pneumococci are isolated from the bloodstream. This is unlikely to represent capsule production during colonization in the nasopharynx, where capsule is less expressed to allow nutrient uptake and adhesion to the mucosal surface. Furthermore it is also difficult to quantify capsule thickness. Working with multiple unknowns will provide less understanding of agglutinating antibodies. By using capsule mutants we aimed to keep constant one condition to be able to measure which protein antigens are suitable for the induction of agglutinating antibodies. There is an essential difference in agglutination between anti-protein and anti-polysaccharide antibodies. Further experiments are needed to confirm the *in vivo* protective effects of these anti-PspA antibodies against colonization with encapsulated *S*. *pneumoniae*.

To summarize, this study demonstrates a novel quantitative, high-throughput technique to determine agglutination of *S*. *pneumoniae*. The efficacy of the pneumococcal conjugate vaccines (PCVs) is correlated with antibody levels [31,32]. The mechanisms by which vaccination-induced antibodies confer protection can differ. For example, opsonophagocytosis is often measured to characterize the functional antibody response [33]. Recently, it was also shown that antibody-mediated agglutination contributes to protection [3,13], which might explain the herd immunity observed upon PCV vaccination [34,35]. However, more in-depth research is needed to confirm a causal relationship in humans. The method presented here will allow high-throughput screening of serum samples to determine the level of agglutinating antibodies. This method is particularly useful to study the contribution of antibody-mediated agglutination to the protection of PCVs. In addition, it may also be used to evaluate novel vaccine candidates.
